# National Trends in Mortality Due to Ischemic Stroke Among Older Adults With Atrial Fibrillation in the USA, 1999–2020

**DOI:** 10.1002/clc.70115

**Published:** 2025-03-15

**Authors:** Saeed Aftab Khan, Arfa Ahmed Assad, Hamza Ashraf, Hanzala Ahmed Farooqi, Sabahat Ul Ain Munir Abbasi, Hira Saleem, Reyan Khalid, Aala Saleh, Muhammad Hashim Akram

**Affiliations:** ^1^ Department of Medicine Allama Iqbal Medical College Lahore Pakistan; ^2^ Islamic International Medical College Riphah International University Rawalpindi Pakistan; ^3^ Faculty of Medicine Lebanese University Beirut Lebanon; ^4^ Chughtai Lab Lahore Pakistan

**Keywords:** atrial fibrillation, CDC WONDER, mortality, older adults, stroke, USA

## Abstract

**Background:**

Atrial fibrillation (AF) is a significant contributor to ischemic stroke risk and mortality, particularly in aging populations. This study examines mortality trends from ischemic stroke secondary to AF in the U.S. from 1999 to 2020, focusing on demographic and regional disparities.

**Methods:**

Using data from the CDC WONDER database, this cross‐sectional analysis included individuals aged ≥ 65 years with death certificates indicating ischemic stroke (ICD I63) and AF (ICD I48) as contributing causes. Age‐adjusted mortality rates (AAMR) were calculated, and temporal trends were analyzed using join‐point regression to estimate annual percentage changes (APC). Data were stratified by age, sex, race/ethnicity, urbanization, and geographic regions.

**Results:**

From 1999 to 2020, ischemic stroke with AF caused 62,443 deaths (AAMR: 6.75/100,000; 95% CI: 6.70–6.80). Mortality rates increased significantly after 2010, peaking between 2014 and 2017 (APC: 31.3 for females, 28.1 for males). Older adults (≥ 85 years) exhibited the highest AAMR (43.2/100,000; 95% CI: 41.6–44.8). Nonmetropolitan areas consistently showed higher mortality compared to metropolitan regions. Demographic disparities were evident, with higher AAMRs in females, Whites, and the Western U.S., though Hispanics had the sharpest APC increase during 2014–2017.

**Conclusion:**

Mortality rates from ischemic stroke with AF are rising in older adults, with significant demographic and regional disparities. The findings underscore the need for targeted public health strategies to mitigate AF‐related stroke risks and improve healthcare equity.

## Graphical abstract

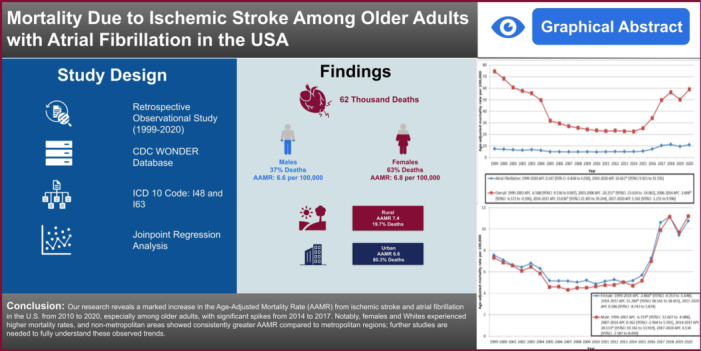

## 1 Introduction

Atrial fibrillation (AF), the most common cardiac arrhythmia, currently affects around 33.5 million people globally [1], with its prevalence expected to double by 2050 [2]. AF is a major contributor to stroke risk, with nearly 25% of ischemic strokes resulting from cardio‐embolism, where AF stands as the primary cause [3]. Nonvalvular AF raises the risk of stroke fivefold [4], while AF associated with mitral stenosis elevates the risk by 20 times [5]. The stroke risk attributable to AF also increases with age, unlike other factors such as hypertension [6]. AF is found in 15% to 38% of ischemic stroke patients, with AF‐related cases resulting in higher complication rates, increased mortality, longer hospital stays, and greater need for nursing care [7, 8, 9].

In the United States, the elderly population is expected to grow from 12.6% in 2000 to 20% by 2030 [10]. This demographic shift is expected to contribute to a rise in the burden of diseases associated with AF, especially strokes. As most stroke survivors live through the initial event but are left with lasting neurological impairments, the resulting disability tends to be long‐term [8, 9]. With the expanding elderly population, AF‐related morbidity and mortality will become increasingly significant in Western societies, making it a crucial topic for discussion. Although there have been published studies reporting trends in AF‐related stroke mortality across different regions of the world [11, 12], there is a notable lack of research focused on disparities in AF‐related stroke mortality in the United States. The aim of this study is to investigate mortality trends in relation to demographic variables, including gender, race or ethnicity, age categories, and geographic regions. By providing current insights that underscore longitudinal trends, this study will contribute to the formulation of effective national plans for preventing and controlling the stroke burden related to AF in the United States.

## 2 Methods

This cross‐sectional analysis utilized mortality data from the Centers for Disease Control and Prevention (CDC) Wide‐ranging On‐line Data for Epidemiological Research database through death certificate queries. Data were collected from the years 1999 to 2020. Death certificates included causes of death in the form of International Classification of Diseases I48 and I63. We queried for all deaths with the underlying causes of death related to ischemic stroke in population age > 65 years. We included all decedents which that reported atrial fibrillation/flutter (ICD: I48 and I63) as the multiple causes of death, or contributors of death. In addition, we obtained overall death rates related to Ischemic stroke without underlying atrial fibrillation as a reference group for comparison.

Death certificate information included demographic information such as age, sex, race and ethnicity, and geographic region. Race and ethnicity were documented by the funeral director as reported by an informant, typically the next of kin. Geographic region was included in death certificate as a component of the US Census Regions (i.e., Northeast, Midwest, South, and West).

Crude death counts and population sizes for each correspondence year were obtained. We adjusted mortality data for age using the direct method, with the year 2000 as the standard US population. The age‐adjusted mortality rates (AAMR) were compared among all sub‐populations based on demographic factors. The lower 95% confidence intervals were calculated by multiplying the crude death rate with the lower 95% confidence limit factor for death rates, which is based on the Poisson variable of the number of deaths. Similarly, the upper 95% confidence interval was obtained by multiplying the crude death rate with the upper 95% confidence limit factor for death rates, also based on the Poisson variable of the number of deaths. To analyze mortality trends over the included 22‐year period, we utilized log‐linear regression models where temporal variation occurred by identifying infection points in mortality trends (Join‐point Regression) [12, 13, 14, 15, 16, 17]. By use of the Monte‐Carlo permutation test, we estimated annual percentage change (APC). Average‐annual percentage change (AAPC) was calculated by taking weighted averages of the APC. Two‐tailed *t*‐test statistics were used to determine statistical significance in increasing or decreasing trends, with *p*‐level set at < 0.05. Due to unreliable data for American Indians, we did not calculate the AAPC for this population. We conducted a comparison between the AAMRs and AAPCs associated with Ischemic stroke with and without underlying atrial fibrillation. To do this, we fitted linear regression lines to the annual changes in AAMR for both groups to ascertain the slopes. Following this, a test of parallelism was performed to establish whether the slopes of the two regression lines were statistically different, both cumulatively and across the various demographic sub‐populations (i.e., sex, urbanization, and US census regions). Institutional Review Board approval was not mandated due to the use of publicly available and identified data. Data visualization and analysis was completed using Stata.

## 3 Results

### 3.1 Overall

**Figure 1:**
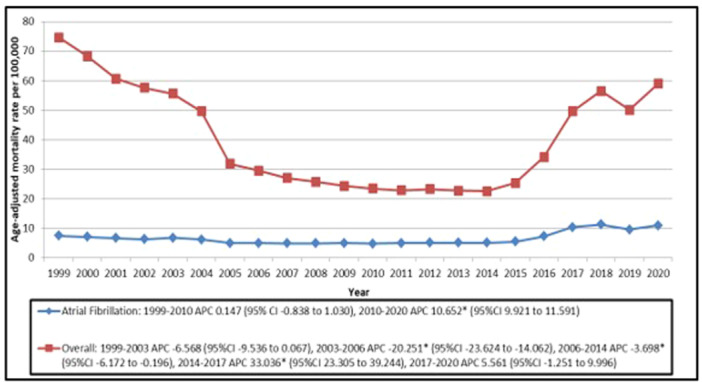
Trends in Age‐adjusted Mortality Rates per 100 000 for Overall Ischemic Stroke and Ischemic Stroke with Atrial Fibrillation in Older Adults the United States from 1999 to 2020. APC indicates Annual Percentage Change. *p < 0.05, 95%CI indicates 95% Confidence Interval.

Ischemic Stroke events secondary to atrial fibrillation were the cause of death of 62,443 people in the United States from 1999 to 2020 (AAMR: 6.75 per 100 000; 95% CI: 6.697–6.803) (Table 1; Figure 1). When stratified by place of death, out of these 62 443 deaths, 31 473 deaths occurred in medical facilities (50.4%), 8661 (13.8%) in decedent's home, 14 558 (23.31%) in nursing home and lastly 5533 (8.86%) in hospice facilities (Table S1).

**Table 1:** Demographic characteristics of deaths due to ischemic stroke among older adults with atrial fibrillation in the USA from 1999 to 2020.

Variable	Ischemic stroke deaths in older adults with atrial fibrillation (n)	AAMR (95% CI) per 100 000
Overall population	62 443 (100%)	6.75 (6.697–6.803)
**Gender** a		
Female	39 375 (63.06%)	6.805 (6.737–6.872)
Male	23 068 (36.94%)	6.598 (6.513–6.684)
**Census Region** a		
Northeast	12 022 (18.3%)	0.784 (0.769–0.798)
Midwest	14 817 (22.5%)	0.884 (0.869–0.898)
South	22 716 (34.6%)	0.877 (0.865–0.888)
West	16 039 (24.5%)	1.039 (1.022–1.055)
**Race/Ethnicity** a		
NH Asian or Pacific Islander	1590 (2.5%)	5.142 (4.887–5.396)
NH Black or African American	3219 (5.18%)	4.385 (4.233–4.538)
NH White	54 647 (88%)	7.185 (7.124–7.245)
Hispanic or Latino	2677 (4.3%)	4.694 (4.515–4.873)
**Urbanization** a		
Metropolitan	50 161 (80.3%)	6.601 (6.543–6.658)
Non metropolitan	12 282 (19.7%)	7.448 (7.316–7.58)
**Place of Death** b		
Medical facility	31 473	—
Decedent's home	8661	—
Hospice facility	5533	—
Nursing home/Long‐term care facility	14 558	—
Other	2131	—

^a^ Age Adjusted Mortality Rates (AAMRs) is utilized.

^b^ Age Adjusted Mortality Rates (AAMRs) is not applicable for Place of Death.

Overall, the AAMR showed a significant increase in AAMR between 2010 and 2020, with an APC of 10.652 (95% CI = 9.921 to 11.591) (Table S2; Figure 1). This increasing trend was most marked between years 2014 and 2017, showing an APC of 31.284 (95% CI = 18.561 to 38.415) and 28.133 (95% CI = 19.342 to 33.919) per 100 000 individuals for women and men respectively (Table S2; Figure 1).

### 3.2 Trends by Gender

**Figure 2:**
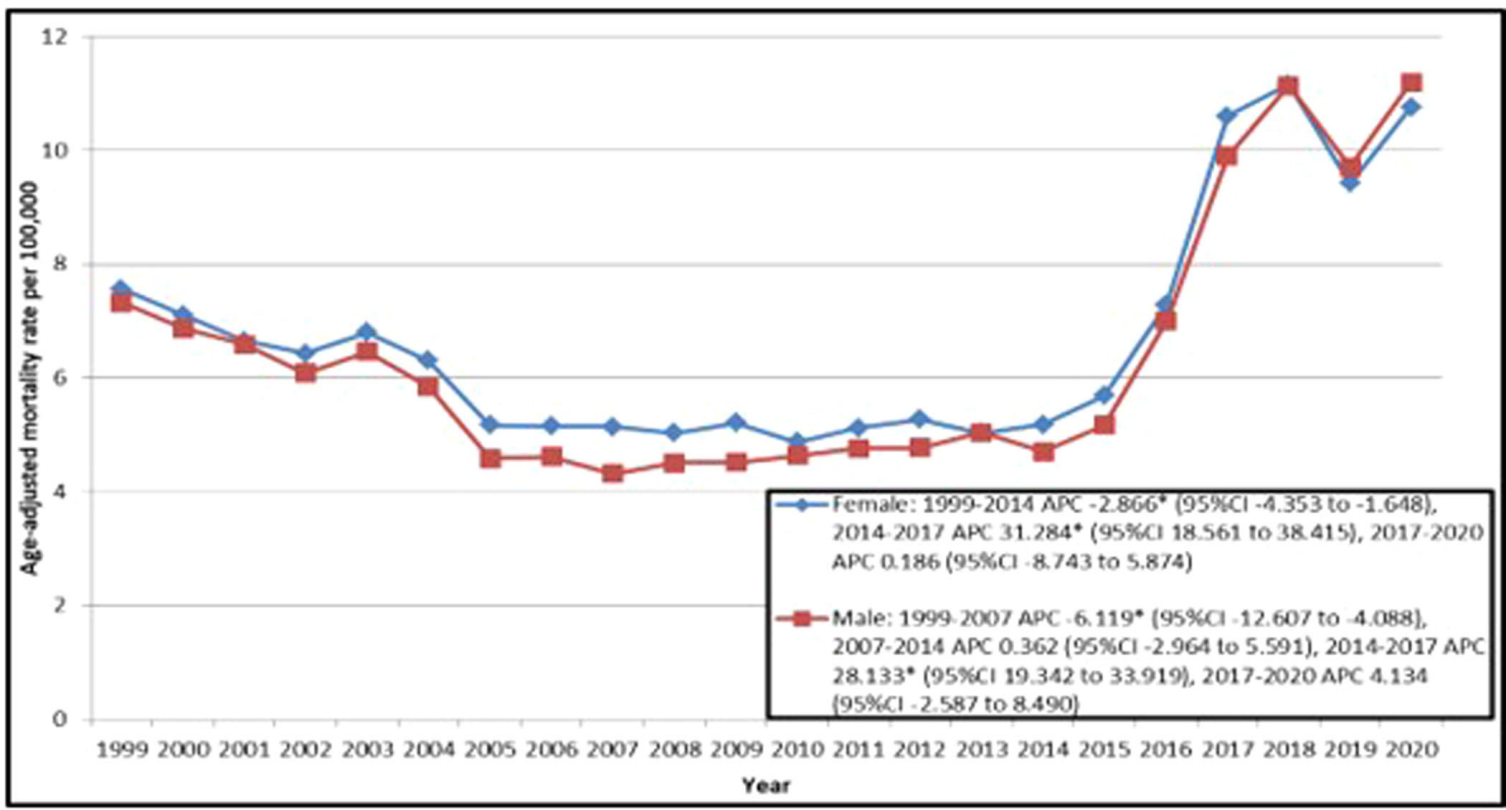
Trends in Ischemic Stroke Age‐adjusted Mortality Rates, Stratified by Gender among Older Adults with Atrial Fibrillation in the United States from 1999 to 2020. APC indicates Annual Percentage Change. *p < 0.05 95%CI indicates 95% Confidence Interval.

Females had more deaths (39 375) than males (23 068) (Table S3; Figure 1) and a higher AAMR of 6.805 (95% CI: 6.737–6.872) versus 6.598 (95% CI: 6.513–6.684) in males (Table 1; Figure 2). The AAMR in females declined significantly from 1692 in 1999 to 1462 in 2014 (APC: −2.866, 95% CI: −4.353 to −1.648) (Table S2; Figure 2), followed by a sharp increase from 2014 to 2017 (APC: 31.284, 95% CI: 18.561–38.415) and a nonsignificant rise from 2017 to 2020 (APC: 0.186, 95% CI: −8.743 to 5.874).

The greatest changes in AAMR occurred between 2014 and 2017 for both genders, with a higher APC increase in females (31.284, 95% CI: 18.561–38.415) than in males (28.133, 95% CI: 19.342–33.919) (Table S2; Figure 2).

### 3.3 Trends by Race/Ethnicity

**Figure 3:**
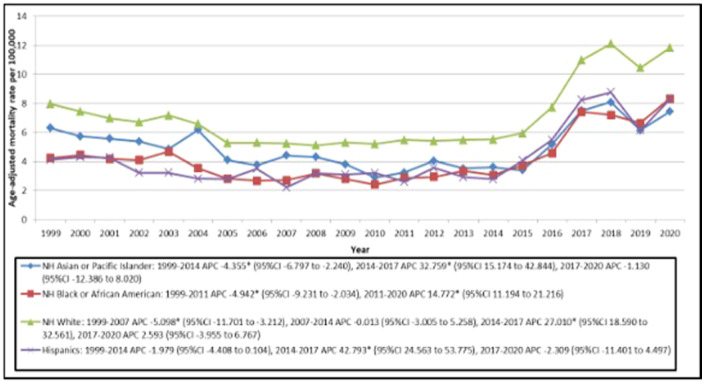
Trends in Ischemic Stroke Age‐adjusted Mortality Rates, Stratified by Race/Ethnicity among Older Adults with Atrial Fibrillation in the United States from 1999 to 2020. APC indicates Annual Percentage Change. *p < 0.05 95%CI indicates 95% Confidence Interval NH indicates Non‐Hispanic.

When stratified by race/ethnicity, White patients reported the highest number of deaths (54 647), followed by Blacks (3219), Hispanics/Latinos (2667) and Asians or Pacific Islanders (1590). The highest AAMR was reported in White populations (7.185; 95% CI = 7.124–7.245), coinciding with the largest number of deaths in the respective population. The second highest AAMR was reported in NH Asians or Pacific Islanders (5.142; 95% CI = 4.887–5.396), followed by Hispanics (4.694; 95% CI = 4.515–4.873) and Blacks (4.385; 95% CI = 4.233–4.538) (Table S4; Figure 3).

Although the White populations had the highest number of deaths and AAMR, the largest increase in AAMR in the period between 2014 and 2017 was seen in Hispanic populations (APC = 42.793; 95% CI = 24.563 to 53.775), followed by NH Whites (APC = 27.010; 95% CI = 18.590 to 32.561) and NH Asians or Pacific Islanders (APC = 32.759; 95% CI = 15.174 to 42.844) respectively. Although a decline in AAMR was noted for Hispanic and NH Asians or Pacific Islander Populations in the years 2017–2020, the APC was not statistically significant (Table S2; Figure 3).

### 3.4 Trends by Urban/Rural Region

**Figure 4:**
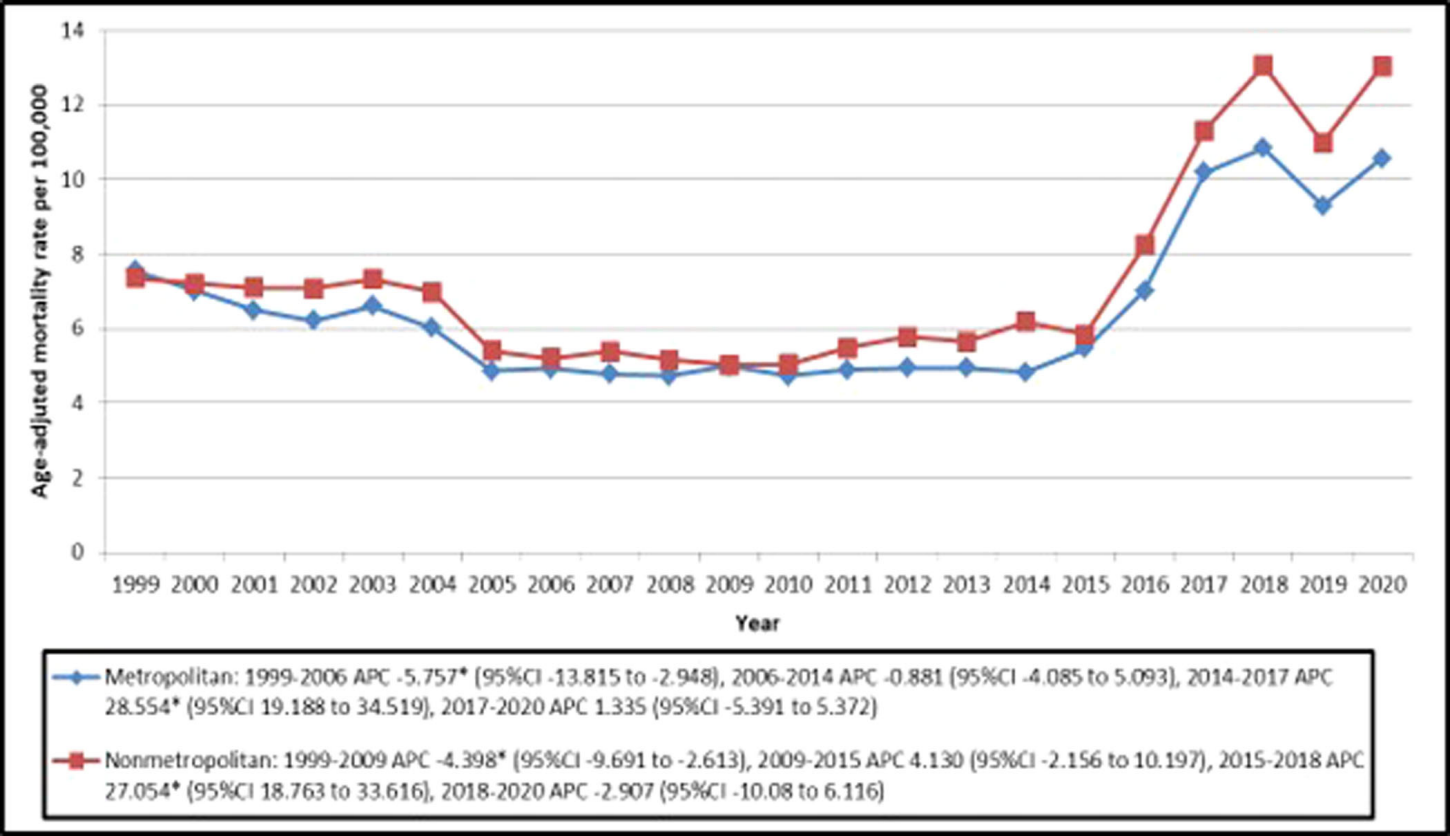
Trends in Ischemic Stroke Age‐adjusted Mortality Rates, Stratified by Urban‐Rural Classification among Older Adults with Atrial Fibrillation in the United States from 1999 to 2020. APC indicates Annual Percentage Change. *p < 0.05. 95%CI indicates 95% Confidence Interval.

From 1999 to 2020, Nonmetropolitan areas had a higher average AAMR (7.448, 95% CI: 7.316–7.580) than metropolitan areas (6.601, 95% CI: 6.543–6.658). In metropolitan areas, AAMR declined steeply from 1999 to 2006 (APC: −5.757, 95% CI: −13.815 to −2.948), remained stable from 2006 to 2014, surged from 2014 to 2017 (APC: 28.554, 95% CI: 19.188–34.519), and showed a moderate increase from 2017 to 2020 (APC: 1.335, 95% CI: −5.391 to 5.372). Nonmetropolitan areas followed a similar pattern, with a significant decline from 1999 to 2009 (APC: −4.398, 95% CI: −9.691 to −2.613), a sharp increase until 2018, and a decline from 2018 to 2020 (APC: −2.907, 95% CI: −10.080 to 6.116) (Table S2; Figure 4).

### 3.5 Trends by Census Region

The West had the highest AAMR (1.039, 95% CI: 1.022–1.055), followed by the Midwest (0.884, 95% CI: 0.869–0.898), the South (0.877, 95% CI: 0.865–0.888), and the Northeast with the lowest (0.784, 95% CI: 0.769–0.798). In the Northeast, AAMR declined from 1999 to 2008 (APC: −5.542, 95% CI: −9.063 to −3.841), increased from 2008 to 2015 (APC: 3.152, 95% CI: −1.817 to 6.886), surged from 2015 to 2018 (APC: 19.639, 95% CI: 12.499–24.713), and declined from 2018 to 2020 (APC: −5.304, 95% CI: −11.800 to 2.816). The Midwest followed a similar trend, with a decline from 1999 to 2010 (APC: −3.713, 95% CI: −9.021 to −1.775), a slight rise until 2015, a sharp increase from 2015 to 2018 (APC: 24.878, 95% CI: 17.065–31.234), and a decline from 2018 to 2020 (APC: −4.705, 95% CI: −11.597 to 4.001). In the South, AAMR decreased from 1999 to 2007 (APC: −5.825, 95% CI: −9.709 to −4.263), remained stable from 2007 to 2014, then rose sharply from 2014 to 2017 (APC: 29.677, 95% CI: 22.468–34.273), continuing to increase from 2017 to 2020. The West showed a decline from 1999 to 2014, followed by a sharp rise from 2014 to 2017 (APC: 36.995, 95% CI: 4.703–47.804), and a slight decline from 2017 to 2020 (Table S2; Figure S1).

### 3.6 Trends by State

Differences in AAMRs were evident across various states. The states with the highest AAMRs—Vermont (AAMR = 16.039; 95% CI = 14.316 to 17.761), Washington (AAMR = 12.97; 95% CI = 12.456 to 13.484), and Oregon (AAMR = 10.7; 95% CI = 10.123 to 11.276)—displayed rates approximately four times higher than those of states at the lower end of the spectrum, such as Louisiana (AAMR = 4.271; 95% CI = 3.903 to 4.639), Nevada (AAMR = 4.583; 95% CI = 4.048 to 5.117), and New York (AAMR = 4.802; 95% CI = 4.631 to 4.974) (Table S5, Figure S2).

### 3.7 Trends by Age Group

Data stratification by age showed the highest AAMR among patients aged 85 and above (AAMR = 43.177; 95%CI = 41.576–44.779), followed by age groups of 75–84 years (AAMR = 10.696; 95% CI = 10.167–11.224) and 65–74 years (AAMR = 2.466; 95% CI = 2.287–2.645) respectively (Table S6, Figure S3).

## 4 Discussion

Our research highlights a significant increase in the Age‐Adjusted Mortality Rate (AAMR) from ischemic stroke and atrial fibrillation in the U.S. between 2010 and 2020 (Table S7), particularly among older age groups, with notable spikes occurring from 2014 to 2017. Females and Whites exhibited higher mortality rates compared to males and other racial groups, respectively. Additionally, nonmetropolitan areas consistently showed a higher AAMR than metropolitan regions (Table S8), and a large proportion of deaths occurred in medical facilities and nursing homes. Overall the western region (Table S9), and particularly the states of Vermont, Washington, and Oregon, reported the highest AAMR, indicating regional disparities in health outcomes related to these conditions.

In our study we found that from 2010 to 2020 the AAMR due to ischemic stroke and AF increased, this rise can be attributed to several interrelated factors. Firstly, the aging population has been associated with worse outcomes in ischemic stroke patients, as older individuals exhibit higher mortality rates and comorbidities that complicate treatment [13, 14]. The increase in age‐adjusted mortality rates from ischemic stroke and atrial fibrillation in the U.S. can be attributed to several interrelated factors. Firstly, advancing age is a significant predictor of poor outcomes following ischemic stroke, with elderly patients experiencing higher mortality and dependency rates compared to younger individuals, independent of stroke severity and complications [15] Additionally, older age correlates with an increased incidence of atrial fibrillation, which is associated with a substantially elevated risk of stroke and mortality [16]. Studies have shown that the presence of comorbidities, such as diabetes and hypertension, further exacerbates the risk of adverse outcomes in older populations [17].

Studies have indicated that older patients often face barriers to accessing timely and effective treatments, which can lead to worse outcomes [18]. Consequently, as the population ages, the prevalence of these conditions rises, leading to an overall increase in age‐adjusted mortality rates from ischemic stroke and atrial fibrillation in the U.S [19]. The findings of these studies align with ours, as our research similarly demonstrates that the Age‐Adjusted Mortality Rate (AAMR) from ischemic stroke and atrial fibrillation rises with increasing age.

Additionally, the prevalence of AF alone also has markedly increased, with studies indicating a fourfold rise in age‐adjusted prevalence and a more than tripling in age‐adjusted incidence rates over decades [20]. This trend suggests that undiagnosed AF may contribute substantially to the stroke burden, as many patients are not identified before their stroke event [21]. Furthermore, acute kidney injury has been linked to higher mortality in stroke patients, indicating that certain comorbid conditions exacerbate the outcomes [22, 23]. Collectively, these factors underscore the complex interplay between demographic changes, disease. prevalence, and healthcare challenges that have led to increased mortality rates during this period. The rise particularly between 2014 and 2017 can be linked to a variety of interconnected factors. For instance, studies have shown that the management of these conditions has not kept pace with their prevalence. Despite advancements in cardiac monitoring leading to increased detection of atrial fibrillation post‐stroke, many patients remain undiagnosed until after an ischemic event [24]. Additionally, the low rates of timely thrombolytic therapy, which is crucial for improving outcomes in ischemic stroke patients, have been a persistent issue [25].

Women experience higher mortality from ischemic stroke and atrial fibrillation due to a combination of biological and systemic factors. Hormonal fluctuations, particularly declining estrogen levels post‐menopause, can enhance prothrombotic states, increasing stroke risk [26, 27]. Structural differences, such as smaller atria and higher atrial fibrosis, may predispose women to more severe arrhythmias and clot formation [28, 29]. Additionally, under‐diagnosis and treatment disparities, including lower anticoagulation rates, contribute to poorer outcomes. Lifestyle factors, such as higher stress levels and lower physical activity, further compound risk [26, 29]. Given that women have greater longevity, they also face a higher lifetime burden of stroke and related comorbidities, exacerbating mortality disparities [30].

Previous studies have also shown that atrial fibrillation (AF) is more prevalent among older White patients, correlating with higher mortality rates post‐stroke [31]. Other studies indicate that the natural history of ischemic stroke in older populations is associated with increased mortality and poorer outcomes, often exacerbated by comorbid conditions prevalent in this demographic [32]. The disparities in stroke incidence and outcomes further highlight that, despite lower incidence rates, the mortality among Whites remains elevated due to these compounded risk factors [33]. AF development is complex, influenced by genetic and environmental factors that remain partly unknown. African Americans appear to have a lower AF risk, while Whites may be more susceptible, possibly due to structural differences like larger left atrial diameter [34]. Studying AF genetics, particularly in African Americans, could reveal novel protective or risk factors. Advances in proteomics and pharmacogenomics highlight racial and gender differences [35], emphasizing the need for diverse enrollment in future research. A deeper molecular understanding of AF may clarify racial disparities and inform targeted therapies [36]. Our study reveals that from 1999 to 2020, nonmetropolitan areas in the U.S. had a higher average Age‐Adjusted Mortality Rate (AAMR) for ischemic stroke and atrial fibrillation (AF) compared to metropolitan areas. This disparity can be attributed to several interconnected factors such as limited healthcare access, delayed medical intervention, and a higher burden of modifiable risk factors in the rural areas. Rural regions often have fewer healthcare providers and stroke centers, leading to delays in diagnosis and treatment [37, 38]. Geographical barriers and transportation issues further contribute to late hospital arrivals, reducing the likelihood of timely thrombolysis or mechanical thrombectomy [38]. Additionally, rural populations exhibit higher rates of smoking, obesity, hypertension, and diabetes, exacerbating stroke risk [39, 40]. Socioeconomic challenges, including lower income levels and reduced health literacy, also hinder preventive care and adherence to treatment [40]. To mitigate these disparities, expanding telehealth services can bridge the gap in specialist care, enabling timely stroke evaluation and AF management [41, 42]. Community‐based interventions, such as targeted health education programs and screening initiatives, can enhance awareness and early detection. Strengthening rural healthcare infrastructure by increasing provider availability, investing in stroke‐ready hospitals, and improving emergency medical services (EMS) response times can further improve outcomes. Addressing these systemic issues is crucial in reducing mortality and ensuring equitable stroke care across all populations [38].

Our findings underscore the urgent need for targeted interventions to mitigate rising mortality from ischemic stroke and atrial fibrillation, particularly among high‐risk populations. Future research should focus on identifying genetic and environmental risk factors contributing to racial and regional disparities in stroke outcomes. Expanding access to specialized stroke care, telemedicine, and early screening programs in underserved areas can significantly reduce mortality. Additionally, improving public awareness, enhancing healthcare infrastructure, and promoting preventive strategies such as lifestyle modifications and better disease management will be crucial in mitigating these trends. A comprehensive, multidisciplinary approach is essential to achieving equitable stroke care and reducing the burden of these conditions nationwide.

### 4.1 Limitations

This study acknowledges several important limitations. Notably, the potential underreporting of conditions such as atrial fibrillation within certain demographic groups poses a challenge to the precision of mortality statistics. Furthermore, the reliance on ICD codes limits the clinical context available, making it difficult to account for comorbidities, disease severity, and specific treatments received—factors that could significantly influence mortality outcomes. Additionally, the absence of data on time from stroke onset to treatment prevents a comprehensive assessment of how delays in medical intervention impact survival rates. The retrospective nature of this research further restricts control over data collection methods, leading to potential gaps in datasets and the presence of unmeasured confounding factors.

Recognizing these limitations is crucial for accurately interpreting our findings and understanding their broader implications. Future research should incorporate more detailed clinical variables, such as treatment patterns and response times, to provide a clearer picture of patient outcomes. Employing a mixed‐methods framework that integrates quantitative data with qualitative insights could further illuminate the underlying causes of health disparities and guide targeted interventions. Additionally, conducting longitudinal studies may help clarify causal relationships and temporal dynamics between atrial fibrillation, ischemic stroke, and mortality. By refining methodological approaches and addressing these data gaps, future studies can generate more precise and actionable insights into mortality trends and healthcare disparities.

## 5 Conclusion

Our research reveals a marked increase in the Age‐Adjusted Mortality Rate (AAMR) from ischemic stroke and atrial fibrillation in the U.S. from 2010 to 2020, especially among older adults, with significant spikes from 2014 to 2017. Notably, females and Whites experienced higher mortality rates, and nonmetropolitan areas showed consistently greater AAMR compared to metropolitan regions; further studies are needed to fully understand these observed trends.

## Author Contributions

**Saeed Aftab Khan:** conceptualization, methodology, project administration, visualization, writing – original draft, writing – review and editing. **Arfa Ahmed Assad:** conceptualization, formal analysis, visualization, writing – original draft, writing – review and editing. **Hamza Ashraf:** project administration, validation, writing – original draft, writing – review and editing. **Hanzala Ahmed Farooqi:** project administration, writing – original draft, writing – review and editing. **Sabahat Ul Ain Munir Abbasi:** writing – original draft, writing – review and editing. **Hira Saleem:** data curation, writing – original draft, writing – review and editing, **Reyan Khalid:** writing – original draft. **Aala Saleh:** writing – original draft, writing – review and editing. **Muhammad Hashim Akram:** writing – original draft, writing – review and editing.

## Conflicts of Interest

The authors declare no conflicts of interest.

## Supporting information

Supporting information.

## Data Availability

The data that support the findings of this study are openly available in CDC WONDER at https://wonder.cdc.gov/, reference number N/A.
