# Supplementary material for: National Trends in Mortality Due to Ischemic Stroke Among Older Adults With Atrial Fibrillation in the USA, 1999–2020
Source: Clin Cardiol. 2025 Mar 15;48(3):e70115. doi: 10.1002/clc.70115 (PMC11909504; doi:10.1002/clc.70115)
Supplement: Supplementary file 1 — Supporting information. [file CLC-48-e70115-s001.docx]

**SUPPLEMENTARY MATERIAL**

| **Deaths** | | | | |  |
| --- | --- | --- | --- | --- | --- |
| **Year** | **Medical facility** | **Nursing home/long-term care facility** | **Hospice Facility** | **Decedent’s Home** | **Other** |
| 1999 | 1403 | 885 | - | 229 | 58 |
| 2000 | 1313 | 857 | - | 224 | 59 |
| 2001 | 1255 | 797 | - | 230 | 63 |
| 2002 | 1204 | 764 | - | 233 | 70 |
| 2003 | 1279 | 813 | - | 254 | 90 |
| 2004 | 1279 | 678 | - | 228 | 85 |
| 2005 | 1152 | 443 | 28 | 168 | 59 |
| 2006 | 1159 | 431 | 55 | 185 | 69 |
| 2007 | 1123 | 441 | 86 | 203 | 55 |
| 2008 | 1146 | 382 | 136 | 195 | 57 |
| 2009 | 1115 | 465 | 140 | 214 | 67 |
| 2010 | 1117 | 391 | 184 | 225 | 73 |
| 2011 | 1146 | 438 | 219 | 262 | 81 |
| 2012 | 1180 | 427 | 263 | 271 | 84 |
| 2013 | 1161 | 468 | 251 | 293 | 106 |
| 2014 | 1175 | 455 | 276 | 310 | 88 |
| 2015 | 1256 | 507 | 370 | 400 | 65 |
| 2016 | 1597 | 700 | 495 | 574 | 89 |
| 2017 | 2590 | 885 | 727 | 741 | 155 |
| 2018 | 2687 | 1099 | 732 | 918 | 207 |
| 2019 | 1985 | 1075 | 773 | 931 | 171 |
| 2020 | 2151 | 1157 | 789 | 1373 | 280 |
| **TOTAL** | 31473 | 14558 | 5533 | 8661 | 2131 |

**Supplementary Table 1: Ischemic Stroke Mortality, Stratified by Place of Death in Older Adults with Atrial Fibrillation in the United States, 1999 to 2020**

**Supplementary Table 2: Annual Percentage Change of Ischemic Stroke Age-Adjusted Mortality Rates per 100,000 in Older Adults with Atrial Fibrillation in the United States, 1999 to 2020**

| Year Interval | APC (95% CI) |
| --- | --- |
| Overall | |
| 1999-2010 | 0.147 (-0.838 to 1.030) |
| 2010-2020 | 10.652* (9.921 to 11.591) |
| Female | |
| 1999-2014 | -2.866* (-4.353 to -1.648) |
| 2014-2017 | 31.284* (18.561 to 38.415) |
| 2017-2020 | 0.186 (-8.743 to 5.874) |
| Male | |
| 1999-2007 | -6.119* (-12.607 to -4.088) |
| 2007-2014 | 0.362 (-2.964 to 5.591) |
| 2014-2017 | 28.133* (19.342 to 33.919) |
| 2017-2020 | 4.134 (-2.587 to 8.490) |
| NH Asian or Pacific Islander | |
| 1999-2014 | -4.355* (-6.797 to -2.240) |
| 2014-2017 | 32.759* (15.174 to 42.844) |
| 2017-2020 | -1.130 (-12.386 to 8.020) |
| NH Black or African American | |
| 1999-2011 | -4.942* (-9.231 to -2.034) |
| 2011-2020 | 14.772* (11.194 to 21.216) |
| NH White | |
| 1999-2007 | -5.098* (-11.701 to -3.212) |
| 2007-2014 | -0.013 (-3.005 to 5.258) |
| 2014-2017 | 27.010* (18.590 to 32.561) |
| 2017-2020 | 2.593 (-3.955 to 6.767) |
| Hispanic or Latino | |
| 1999-2014 | -1.979 (-4.408 to 0.104) |
| 2014-2017 | 42.793* (24.563 to 53.775) |
| 2017-2020 | -2.309 (-11.401 to 4.497) |
| Metropolitan | |
| 1999-2006 | -5.757* (-13.815 to -2.948) |
| 2006-2014 | -0.881 (-4.085 to 5.093) |
| 2014-2017 | 28.554* (19.188 to 34.519) |
| 2017-2020 | 1.335 (-5.391 to 5.372) |
| Non-Metropolitan | |
| 1999-2009 | -4.398* (-9.691 to -2.613) |
| 2009-2015 | 4.130 (-2.156 to 10.197) |
| 2015-2018 | 27.054* (18.763 to 33.616) |
| 2018-2020 | -2.907 (-10.08 to 6.116) |
| Northeast | |
| 1999-2008 | -5.542*(-9.0632 to -3.8414) |
| 2008-2015 | 3.1521(-1.8165 to 6.8856) |
| 2015-2018 | 19.6385*(12.4990 to 24.7128) |
| 2018-2020 | -5.3036(-11.7997 to 2.8157) |
| Midwest | |
| 1999-2010 | -3.7127*( -9.0206 to -1.7747) |
| 2010-2015 | 3.4445(-3.5122 to 9.1857) |
| 2015-2018 | 24.8775*(17.0647 to 31.2343) |
| 2018-2020 | -4.7050(-11.5974 to 4.0014) |
| South | |
| 1999-2007 | -5.8246*( -9.7087 to -4.2630) |
| 2007-2014 | -0.1363(-2.6222 to 3.7383) |
| 2014-2017 | 29.6767*(22.4677 to 34.2733) |
| 2017-2020 | 4.6643*(0.3632 to 8.0221) |
| West | |
| 1999-2014 | -3.1893*( -5.7437 to -1.0805) |
| 2014-2017 | 36.9950*(4.7025 to 47.8043) |
| 2017-2020 | -1.9773(-14.6227 to 10.3085) |

APC= Annual Percentage Change; AAMR= Age-adjusted mortality rate; NH= Non –Hispanic; *P<0.05

**Supplementary Table 3: Ischemic Stroke Mortality in Older Adults with Atrial Fibrillation in the United States, 1999 to 2020**

| **Year** | **Overall** | **Females** | **Males** | **Asian or Pacific Islander** | **Black or African American** | **White** | **Hispanic** | **Total Population** |
| --- | --- | --- | --- | --- | --- | --- | --- | --- |
| 1999 | 2576 | 1692 | 884 | 39 | 113 | 2363 | 56 | 34797841 |
| 2000 | 2454 | 1613 | 841 | 39 | 118 | 2221 | 63 | 34991753 |
| 2001 | 2345 | 1520 | 825 | 43 | 112 | 2116 | 63 | 35290291 |
| 2002 | 2271 | 1495 | 776 | 40 | 109 | 2060 | 52 | 35522207 |
| 2003 | 2449 | 1604 | 845 | 40 | 127 | 2224 | 53 | 35863529 |
| 2004 | 2282 | 1501 | 781 | 53 | 99 | 2072 | 49 | 36203319 |
| 2005 | 1857 | 1229 | 628 | 40 | 81 | 1679 | 52 | 36649798 |
| 2006 | 1904 | 1246 | 658 | 38 | 78 | 1711 | 68 | 37164107 |
| 2007 | 1908 | 1282 | 626 | 48 | 80 | 1729 | 46 | 37825711 |
| 2008 | 1937 | 1269 | 668 | 48 | 97 | 1716 | 69 | 38777621 |
| 2009 | 2029 | 1339 | 690 | 47 | 88 | 1811 | 74 | 39623175 |
| 2010 | 1991 | 1270 | 721 | 37 | 76 | 1792 | 77 | 40267984 |
| 2011 | 2147 | 1377 | 770 | 45 | 96 | 1928 | 69 | 41394141 |
| 2012 | 2225 | 1428 | 797 | 60 | 103 | 1952 | 101 | 43145356 |
| 2013 | 2281 | 1404 | 877 | 58 | 120 | 2002 | 87 | 44704074 |
| 2014 | 2306 | 1462 | 844 | 64 | 111 | 2034 | 86 | 46243211 |
| 2015 | 2598 | 1646 | 952 | 64 | 142 | 2231 | 138 | 47760852 |
| 2016 | 3456 | 2134 | 1322 | 106 | 180 | 2952 | 197 | 49244195 |
| 2017 | 5098 | 3148 | 1950 | 163 | 302 | 4283 | 313 | 50858679 |
| 2018 | 5644 | 3406 | 2238 | 182 | 306 | 4774 | 352 | 52431193 |
| 2019 | 4935 | 2934 | 2001 | 148 | 296 | 4202 | 254 | 54058263 |
| 2020 | 5750 | 3376 | 2374 | 188 | 385 | 4795 | 358 | 55659365 |
| **Total** | 62443 | 39375 | 23068 | 1590 | 3219 | 54647 | 2677 | 9.28E+08 |
| **Year** | **Females** | **Males** |  |  |  |  |  |  |
| 1999 | 7.562 (7.201-7.924) | 7.318 (6.827-7.81) |  |  |  |  |  |  |
| 2000 | 7.105 (6.757-7.453) | 6.862 (6.39-7.334) |  |  |  |  |  |  |
| 2001 | 6.651 (6.315-6.987) | 6.584 (6.128-7.04) |  |  |  |  |  |  |
| 2002 | 6.433 (6.106-6.761) | 6.078 (5.644-6.513) |  |  |  |  |  |  |
| 2003 | 6.801 (6.467-7.136) | 6.46 (6.018-6.901) |  |  |  |  |  |  |
| 2004 | 6.302 (5.982-6.622) | 5.846 (5.431-6.261) |  |  |  |  |  |  |
| 2005 | 5.17 (4.879-5.461) | 4.584 (4.222-4.946) |  |  |  |  |  |  |
| 2006 | 5.148 (4.86-5.437) | 4.608 (4.253-4.962) |  |  |  |  |  |  |
| 2007 | 5.135 (4.852-5.419) | 4.317 (3.977-4.658) |  |  |  |  |  |  |
| 2008 | 5.031 (4.751-5.311) | 4.502 (4.159-4.845) |  |  |  |  |  |  |
| 2009 | 5.204 (4.922-5.487) | 4.517 (4.179-4.856) |  |  |  |  |  |  |
| 2010 | 4.868 (4.596-5.139) | 4.632 (4.292-4.971) |  |  |  |  |  |  |
| 2011 | 5.12 (4.845-5.395) | 4.761 (4.423-5.098) |  |  |  |  |  |  |
| 2012 | 5.269 (4.991-5.547) | 4.773 (4.44-5.106) |  |  |  |  |  |  |
| 2013 | 5.026 (4.758-5.294) | 5.039 (4.703-5.375) |  |  |  |  |  |  |
| 2014 | 5.175 (4.905-5.446) | 4.703 (4.383-5.022) |  |  |  |  |  |  |
| 2015 | 5.684 (5.404-5.963) | 5.172 (4.841-5.503) |  |  |  |  |  |  |
| 2016 | 7.284 (6.969-7.598) | 6.994 (6.614-7.374) |  |  |  |  |  |  |
| 2017 | 10.603 (10.227-10.98) | 9.898 (9.455-10.342) |  |  |  |  |  |  |
| 2018 | 11.154 (10.775-11.534) | 11.132 (10.667-11.596) |  |  |  |  |  |  |
| 2019 | 9.421 (9.077-9.766) | 9.705 (9.277-10.134) |  |  |  |  |  |  |
| 2020 | 10.755 (10.389-11.12) | 11.19 (10.737-11.644) |  |  |  |  |  |  |
| Total | 6.805 (6.737-6.872) | 6.598 (6.513-6.684) |  |  |  |  |  |  |

**Supplementary Table 4: Ischemic Stroke Age-Adjusted Mortality Rates per 100,000, Stratified by Race/Ethnicity in Older Adults with Atrial Fibrillation in the United States, 1999 to 2020**

| **Age-adjusted mortality rates (95% CI)** | | | | |
| --- | --- | --- | --- | --- |
| **Year** | **NH white** | **NH Black or African American** | **Hispanic or Latino** | **NH Asian or Pacific Islander** |
| 1999 | 7.956 (7.635-8.277) | 4.242 (3.457-5.027) | 4.115 (3.091-5.369) | 6.313 (4.445-8.702) |
| 2000 | 7.438 (7.129-7.748) | 4.455 (3.649-5.261) | 4.31 (3.297-5.536) | 5.732 (4.036-7.901) |
| 2001 | 6.974 (6.677-7.271) | 4.193 (3.414-4.971) | 4.272 (3.268-5.488) | 5.578 (4.003-7.567) |
| 2002 | 6.707 (6.417-6.997) | 4.102 (3.33-4.874) | 3.223 (2.392-4.249) | 5.385 (3.829-7.361) |
| 2003 | 7.174 (6.875-7.472) | 4.682 (3.865-5.5) | 3.232 (2.414-4.239) | 4.847 (3.447-6.626) |
| 2004 | 6.597 (6.313-6.882) | 3.541 (2.875-4.315) | 2.83 (2.08-3.764) | 6.167 (4.606-8.088) |
| 2005 | 5.279 (5.026-5.532) | 2.826 (2.241-3.517) | 2.81 (2.092-3.695) | 4.108 (2.921-5.616) |
| 2006 | 5.295 (5.043-5.546) | 2.687 (2.12-3.358) | 3.501 (2.713-4.446) | 3.746 (2.637-5.163) |
| 2007 | 5.235 (4.988-5.483) | 2.715 (2.149-3.383) | 2.236 (1.631-2.992) | 4.416 (3.245-5.873) |
| 2008 | 5.112 (4.869-5.355) | 3.199 (2.591-3.906) | 3.172 (2.463-4.022) | 4.317 (3.183-5.724) |
| 2009 | 5.314 (5.068-5.56) | 2.802 (2.244-3.457) | 3.11 (2.438-3.91) | 3.796 (2.779-5.063) |
| 2010 | 5.2 (4.958-5.442) | 2.417 (1.901-3.03) | 3.204 (2.525-4.011) | 2.912 (2.04-4.032) |
| 2011 | 5.492 (5.245-5.739) | 2.869 (2.322-3.508) | 2.613 (2.029-3.312) | 3.247 (2.36-4.359) |
| 2012 | 5.412 (5.17-5.655) | 2.952 (2.378-3.526) | 3.562 (2.863-4.261) | 4.053 (3.093-5.217) |
| 2013 | 5.484 (5.241-5.727) | 3.354 (2.748-3.96) | 2.932 (2.345-3.621) | 3.524 (2.669-4.566) |
| 2014 | 5.525 (5.282-5.767) | 3.071 (2.495-3.647) | 2.787 (2.227-3.447) | 3.605 (2.771-4.613) |
| 2015 | 5.945 (5.696-6.195) | 3.732 (3.112-4.352) | 4.071 (3.389-4.754) | 3.405 (2.617-4.356) |
| 2016 | 7.735 (7.453-8.016) | 4.582 (3.905-5.259) | 5.488 (4.715-6.261) | 5.173 (4.181-6.164) |
| 2017 | 10.975 (10.643-11.307) | 7.405 (6.56-8.249) | 8.242 (7.322-9.162) | 7.494 (6.335-8.653) |
| 2018 | 12.085 (11.74-12.43) | 7.197 (6.382-8.013) | 8.753 (7.831-9.675) | 8.076 (6.895-9.257) |
| 2019 | 10.447 (10.13-10.765) | 6.645 (5.877-7.412) | 6.141 (5.38-6.902) | 6.146 (5.149-7.143) |
| 2020 | 11.813 (11.478-12.149) | 8.323 (7.479-9.166) | 8.264 (7.402-9.126) | 7.424 (6.356-8.493) |
| TOTAL | 7.185 (7.124-7.245) | 4.385 (4.233-4.538) | 4.694 (4.515-4.873) | 5.142 (4.887-5.396) |

**Supplementary Table 5: Ischemic Stroke Age-Adjusted Mortality Rates per 100,000, Stratified by States in Older Adults with Atrial Fibrillation in the United States, 1999 to 2020**

| **State** | **Age-adjusted Rate (95% CI)** |
| --- | --- |
| Alabama | 5.415 (5.028-5.801) |
| Alaska | 8.859 (7.066-10.968) |
| Arizona | 7.244 (6.862-7.626) |
| Arkansas | 6.702 (6.171-7.233) |
| California | 7.019 (6.853-7.184) |
| Colorado | 7.348 (6.863-7.833) |
| Connecticut | 5.939 (5.514-6.364) |
| Delaware | 5.971 (5.059-6.883) |
| District of Columbia | 6.462 (5.23-7.694) |
| Florida | 4.902 (4.744-5.059) |
| Georgia | 5.168 (4.862-5.474) |
| Hawaii | 9.3 (8.42-10.179) |
| Idaho | 10.559 (9.58-11.537) |
| Illinois | 5.057 (4.83-5.283) |
| Indiana | 6.738 (6.369-7.106) |
| Iowa | 8.488 (7.952-9.024) |
| Kansas | 5.563 (5.084-6.042) |
| Kentucky | 6.926 (6.462-7.391) |
| Louisiana | 4.271 (3.903-4.639) |
| Maine | 8.62 (7.791-9.448) |
| Maryland | 7.556 (7.13-7.982) |
| Massachusetts | 5.554 (5.243-5.864) |
| Michigan | 6.573 (6.288-6.858) |
| Minnesota | 9.279 (8.815-9.742) |
| Mississippi | 5.323 (4.823-5.823) |
| Missouri | 5.231 (4.907-5.556) |
| Montana | 6.469 (5.597-7.341) |
| Nebraska | 5.607 (5.011-6.203) |
| Nevada | 4.583 (4.048-5.117) |
| New Hampshire | 10.441 (9.451-11.431) |
| New Jersey | 4.933 (4.673-5.193) |
| New Mexico | 5.197 (4.608-5.787) |
| New York | 4.802 (4.631-4.974) |
| North Carolina | 7.558 (7.224-7.891) |
| North Dakota | 7.509 (6.439-8.579) |
| Ohio | 7.152 (6.882-7.423) |
| Oklahoma | 6.138 (5.677-6.599) |
| Oregon | 10.7 (10.123-11.276) |
| Pennsylvania | 7.703 (7.454-7.953) |
| Rhode Island | 5.89 (5.126-6.654) |
| South Carolina | 8.471 (7.969-8.974) |
| South Dakota | 7.259 (6.275-8.242) |
| Tennessee | 9.002 (8.564-9.44) |
| Texas | 8.439 (8.198-8.681) |
| Utah | 6.389 (5.712-7.067) |
| Vermont | 16.039 (14.316-17.761) |
| Virginia | 6.315 (5.976-6.653) |
| Washington | 12.97 (12.456-13.484) |
| West Virginia | 6.901 (6.26-7.542) |
| Wisconsin | 7.295 (6.908-7.682) |
| Wyoming | 6.336 (5.132-7.737) |

**Supplementary Table 6: Ischemic Stroke Crude Mortality Rates, Stratified by Ten-Year Age Groups in Older Adults with Atrial Fibrillation in the United States, 1999 to 2020**

| **Year** | **65-74 years** | **75-84 years** | **85+ years** |
| --- | --- | --- | --- |
| 1999 | 1.607(1.424-1.79) | 8.524(8.006-9.041) | 29.802(28.142-31.463) |
| 2000 | 1.484(1.308-1.661) | 7.629(7.142-8.116) | 29.201(27.574-30.828) |
| 2001 | 1.354(1.186-1.523) | 7.401(6.925-7.876) | 26.991(25.441-28.542) |
| 2002 | 1.338(1.171-1.505) | 7.035(6.575-7.495) | 25.797(24.29-27.303) |
| 2003 | 1.405(1.235-1.576) | 7.18(6.718-7.643) | 28.279(26.72-29.839) |
| 2004 | 1.27(1.108-1.431) | 6.682(6.238-7.127) | 25.892(24.412-27.371) |
| 2005 | 1.117(0.967-1.268) | 5.66(5.252-6.068) | 19.304(18.047-20.561) |
| 2006 | 1.182(1.028-1.336) | 5.659(5.251-6.066) | 19.236(18.003-20.468) |
| 2007 | 1.081(0.936-1.227) | 5.219(4.827-5.61) | 20.081(18.844-21.318) |
| 2008 | 0.98(0.845-1.116) | 5.254(4.861-5.647) | 20.189(18.967-21.411) |
| 2009 | 1.041(0.904-1.178) | 5.606(5.199-6.012) | 20.085(18.886-21.284) |
| 2010 | 1.05(0.914-1.186) | 5.183(4.793-5.574) | 19.769(18.593-20.945) |
| 2011 | 1.143(1.003-1.283) | 5.442(5.044-5.84) | 20.446(19.276-21.616) |
| 2012 | 1.038(0.909-1.167) | 5.568(5.166-5.969) | 21.011(19.84-22.182) |
| 2013 | 1.178(1.044-1.312) | 5.347(4.956-5.738) | 20.941(19.787-22.095) |
| 2014 | 1.011(0.89-1.133) | 5.671(5.272-6.07) | 20.496(19.365-21.626) |
| 2015 | 1.234(1.103-1.365) | 5.904(5.5-6.307) | 22.84(21.659-24.022) |
| 2016 | 1.575(1.43-1.721) | 7.679(7.224-8.134) | 29.967(28.624-31.31) |
| 2017 | 2.466(2.287-2.645) | 10.696(10.167-11.224) | 43.177(41.576-44.779) |
| 2018 | 2.46(2.284-2.636) | 11.764(11.222-12.306) | 47.108(45.445-48.771) |
| 2019 | 2.134(1.973-2.296) | 9.737(9.253-10.221) | 41(39.455-42.544) |
| 2020 | 2.436(2.267-2.606) | 11.658(11.137-12.18) | 45.641(44.019-47.264) |
| Total | 1.503(1.469-1.536) | 7.233(7.136-7.329) | 27.765(27.466-28.064) |

**Supplementary Table 7: Ischemic Stroke Age-Adjusted Mortality Rates, Overall and in those with Atrial Fibrillation in Older Adults in the United States, 1999-2020**

| **Year** | **Overall** | **Atrial Fibrillation** |
| --- | --- | --- |
| 1999 | 74.674(73.759-75.588) | 7.508(7.219-7.798) |
| 2000 | 68.224(67.356-69.092) | 7.063(6.784-7.343) |
| 2001 | 60.68(59.867-61.492) | 6.67(6.4-6.94) |
| 2002 | 57.577(56.79-58.364) | 6.329(6.068-6.589) |
| 2003 | 55.575(54.808-56.343) | 6.759(6.491-7.026) |
| 2004 | 49.562(48.841-50.283) | 6.235(5.979-6.49) |
| 2005 | 31.893(31.32-32.467) | 4.965(4.739-5.191) |
| 2006 | 29.53(28.984-30.076) | 5.005(4.78-5.23) |
| 2007 | 27.034(26.516-27.552) | 4.886(4.667-5.106) |
| 2008 | 25.722(25.221-26.222) | 4.882(4.664-5.1) |
| 2009 | 24.377(23.893-24.861) | 4.976(4.759-5.193) |
| 2010 | 23.508(23.037-23.979) | 4.849(4.635-5.063) |
| 2011 | 22.858(22.399-23.317) | 4.994(4.781-5.206) |
| 2012 | 23.228(22.772-23.685) | 5.086(4.873-5.299) |
| 2013 | 22.727(22.281-23.173) | 5.072(4.862-5.282) |
| 2014 | 22.598(22.159-23.037) | 5.06(4.852-5.269) |
| 2015 | 25.406(24.945-25.867) | 5.518(5.304-5.732) |
| 2016 | 34.166(33.638-34.694) | 7.249(7.005-7.493) |
| 2017 | 49.567(48.939-50.194) | 10.404(10.116-10.691) |
| 2018 | 56.537(55.877-57.197) | 11.272(10.977-11.568) |
| 2019 | 50.117(49.504-50.731) | 9.57(9.301-9.838) |
| 2020 | 58.945(58.286-59.604) | 11.001(10.715-11.286) |
| Total | 40.254(40.124-40.384) | 6.75(6.697-6.803) |

**Supplementary Table 8: Ischemic Stroke Age-Adjusted Mortality Rates per 100,000, Stratified by Urban-Rural Classification in Older Adults with Atrial Fibrillation in the United States, 1999 to 2020**

| Year | **Metropolitan** | **Nonmetropolitan** |
| --- | --- | --- |
| 1999 | 7.558 (7.233-7.882) | 7.366 (6.717-8.016) |
| 2000 | 7.023 (6.713-7.334) | 7.225 (6.585-7.866) |
| 2001 | 6.509 (6.213-6.805) | 7.107 (6.474-7.739) |
| 2002 | 6.218 (5.93-6.507) | 7.086 (6.454-7.718) |
| 2003 | 6.608 (6.314-6.902) | 7.331 (6.692-7.97) |
| 2004 | 6.035 (5.756-6.314) | 6.994 (6.371-7.617) |
| 2005 | 4.87 (4.622-5.118) | 5.404 (4.858-5.951) |
| 2006 | 4.939 (4.692-5.187) | 5.218 (4.686-5.75) |
| 2007 | 4.789 (4.548-5.03) | 5.392 (4.854-5.93) |
| 2008 | 4.745 (4.509-4.981) | 5.166 (4.644-5.687) |
| 2009 | 4.999 (4.758-5.24) | 5.034 (4.519-5.549) |
| 2010 | 4.738 (4.506-4.97) | 5.052 (4.539-5.564) |
| 2011 | 4.911 (4.678-5.144) | 5.498 (4.967-6.028) |
| 2012 | 4.954 (4.722-5.186) | 5.787 (5.248-6.326) |
| 2013 | 4.958 (4.73-5.187) | 5.65 (5.122-6.178) |
| 2014 | 4.843 (4.619-5.068) | 6.191 (5.639-6.742) |
| 2015 | 5.469 (5.234-5.704) | 5.846 (5.319-6.373) |
| 2016 | 7.018 (6.755-7.281) | 8.252 (7.623-8.88) |
| 2017 | 10.197 (9.885-10.51) | 11.325 (10.598-12.051) |
| 2018 | 10.854 (10.536-11.172) | 13.065 (12.294-13.836) |
| 2019 | 9.313 (9.022-9.604) | 11.002 (10.3-11.703) |
| 2020 | 10.576 (10.269-10.883) | 13.048 (12.287-13.808) |
| Total | 6.601 (6.543-6.658) | 7.448 (7.316-7.58) |

**Supplementary Table 9: Ischemic Stroke Age-Adjusted Mortality Rates per 100,000, Stratified by Census Regions in Older Adults with Atrial Fibrillation in the United States, 1999 to 2020**

| **Year** | **Northeast** | **Midwest** | **South** | **West** |
| --- | --- | --- | --- | --- |
| 1999 | 0.934 (0.856-1.013) | 0.983 (0.907-1.059) | 0.936 (0.874-0.998) | 1.118 (1.028-1.209) |
| 2000 | 0.843 (0.77-0.917) | 0.862 (0.791-0.932) | 0.89 (0.83-0.95) | 1.074 (0.986-1.162) |
| 2001 | 0.853 (0.779-0.927) | 0.849 (0.779-0.919) | 0.835 (0.778-0.893) | 0.971 (0.889-1.054) |
| 2002 | 0.801 (0.73-0.872) | 0.803 (0.736-0.87) | 0.813 (0.756-0.869) | 0.899 (0.821-0.977) |
| 2003 | 0.799 (0.729-0.87) | 0.864 (0.795-0.934) | 0.848 (0.791-0.906) | 1.041 (0.958-1.124) |
| 2004 | 0.792 (0.722-0.862) | 0.79 (0.724-0.856) | 0.719 (0.667-0.772) | 1.008 (0.926-1.09) |
| 2005 | 0.658 (0.595-0.722) | 0.728 (0.665-0.792) | 0.587 (0.54-0.633) | 0.653 (0.588-0.717) |
| 2006 | 0.617 (0.556-0.678) | 0.676 (0.616-0.736) | 0.632 (0.583-0.68) | 0.716 (0.65-0.783) |
| 2007 | 0.567 (0.509-0.625) | 0.693 (0.632-0.754) | 0.592 (0.546-0.638) | 0.723 (0.656-0.789) |
| 2008 | 0.559 (0.502-0.616) | 0.699 (0.638-0.76) | 0.569 (0.525-0.614) | 0.726 (0.661-0.791) |
| 2009 | 0.583 (0.525-0.64) | 0.647 (0.589-0.705) | 0.625 (0.579-0.671) | 0.772 (0.706-0.838) |
| 2010 | 0.604 (0.545-0.662) | 0.619 (0.563-0.676) | 0.585 (0.54-0.629) | 0.728 (0.664-0.792) |
| 2011 | 0.562 (0.506-0.618) | 0.688 (0.629-0.746) | 0.613 (0.569-0.658) | 0.808 (0.742-0.874) |
| 2012 | 0.645 (0.585-0.705) | 0.634 (0.579-0.69) | 0.641 (0.596-0.686) | 0.787 (0.722-0.851) |
| 2013 | 0.679 (0.618-0.74) | 0.674 (0.617-0.731) | 0.613 (0.57-0.657) | 0.765 (0.702-0.827) |
| 2014 | 0.671 (0.611-0.732) | 0.758 (0.697-0.819) | 0.605 (0.563-0.648) | 0.675 (0.616-0.733) |
| 2015 | 0.706 (0.644-0.768) | 0.706 (0.648-0.764) | 0.695 (0.65-0.74) | 0.803 (0.741-0.865) |
| 2016 | 0.837 (0.77-0.904) | 0.907 (0.842-0.972) | 0.97 (0.918-1.022) | 1.121 (1.049-1.193) |
| 2017 | 1.115 (1.039-1.19) | 1.312 (1.235-1.39) | 1.34 (1.28-1.401) | 1.736 (1.647-1.826) |
| 2018 | 1.241 (1.162-1.32) | 1.434 (1.354-1.513) | 1.464 (1.401-1.526) | 1.844 (1.754-1.935) |
| 2019 | 1.023 (0.951-1.095) | 1.204 (1.132-1.277) | 1.35 (1.291-1.408) | 1.417 (1.339-1.495) |
| 2020 | 1.173 (1.097-1.249) | 1.399 (1.321-1.477) | 1.544 (1.481-1.606) | 1.653 (1.57-1.737) |
| Total | 0.784 (0.769-0.798) | 0.884 (0.869-0.898) | 0.877 (0.865-0.888) | 1.039 (1.022-1.055) |

**
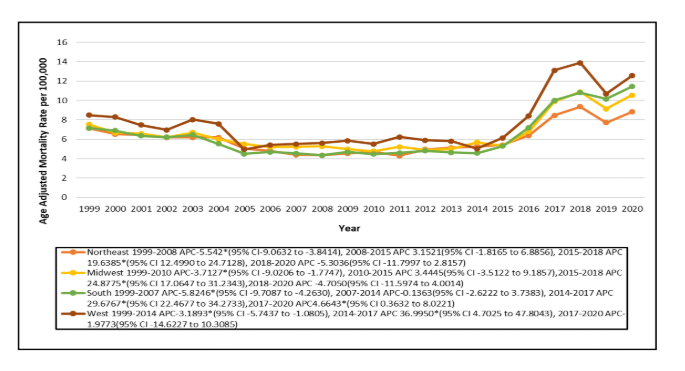
**

***Supplementary Figure 1:*** Trends in Ischemic Stroke Age-adjusted Mortality Rates, Stratified by Census Regions among Older Adults with Atrial Fibrillation in the United States from 1999-2020

APC indicates Annual Percentage Change. *P<0.05

95%CI indicates 95% Confidence Interval

**
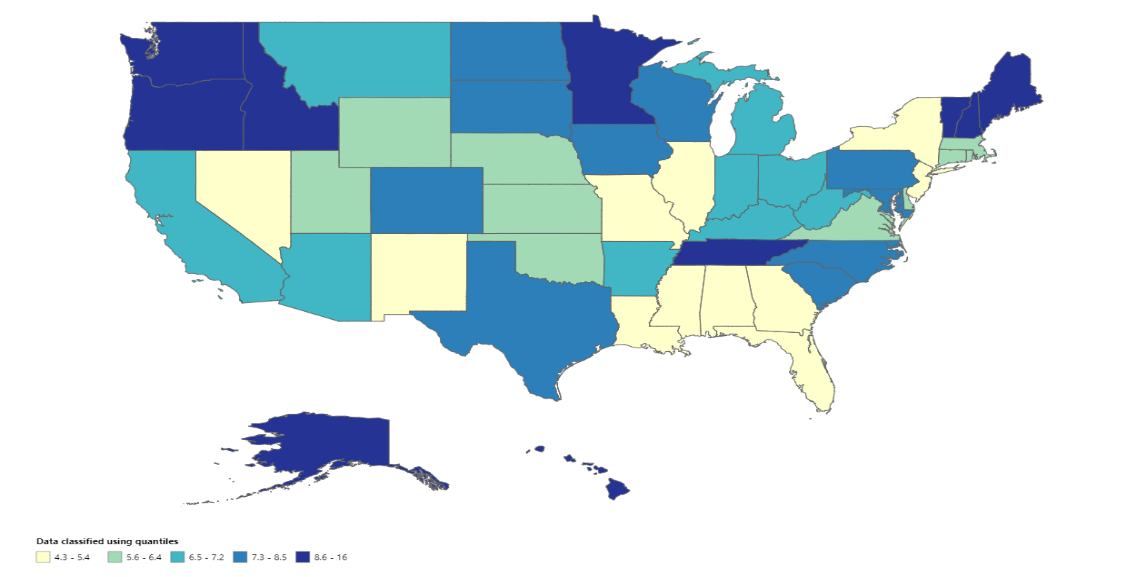
**

***Supplementary Figure 2:*** USA Map showing Age-adjusted mortality rates per 100,000 across different states

**
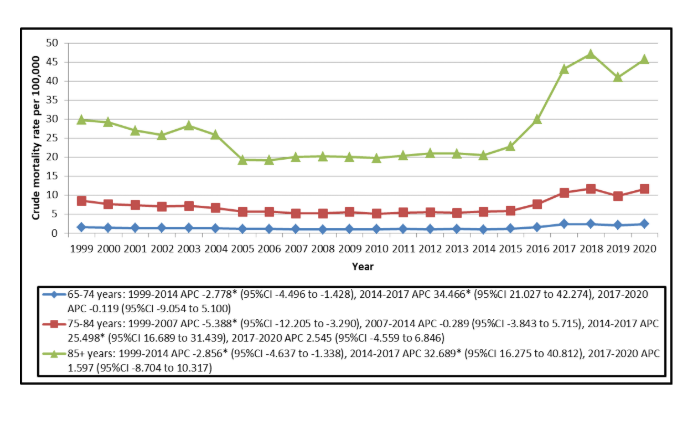
**

**Supplementary Figure 3** Trends in Ischemic Stroke Crude Mortality Rates, Stratified by Ten-Year Age Groups among Older Adults with Atrial Fibrillation in the United States from 1999-2020

APC indicates Annual Percentage Change. *P<0.05

95%CI indicates 95% Confidence Interval
